# Genomics reveal population structure, genetic diversity and evolutionary history of *Phyllostachys edulis* (moso bamboo) in global natural distribution

**DOI:** 10.3389/fpls.2025.1532058

**Published:** 2025-05-15

**Authors:** Fangdi Li, Hongfeng Fang, Jie Zhou, Shunkai Hu, Fuliang Cao, Qirong Guo

**Affiliations:** ^1^ Co-Innovation Center for Sustainable Forestry in Southern China, College of Forestry and Grassland College of Soil and Water Conservation, Nanjing Forestry University, Nanjing, China; ^2^ Zhejiang Modern State-Owned Forest Farm, Longshan Forest Farm, Anji, Zhejiang, China

**Keywords:** *Phyllostachys edulis*, GBS, population structure, genetic variation, germplasm resources

## Abstract

**Introduction:**

Moso bamboo (*Phyllostachys edulis*) is widespread in natural forests over large areas in China

**Methods:**

Here we collected 193 individuals of moso bamboo from 37 natural populations in China’s distribution area. Genotyping by sequencing (GBS) was employed to elucidate the genetic diversity, genetic structure, selection pressure, history and adaptive distribution prediction of moso bamboo.

**Results:**

The results revealed that the moso bamboo in China can be divided into central α, eastern β and southern γ subpopulations, with the α-subpopulation presumed to be the origin center. Notably, the genetic diversity of moso bamboo populations were relatively low, and the heterozygotes were excess. At the subpopulation level, the genetic diversity of α-subpopulation was the highest and that of β-subpopulation was the lowest. Analysis of population selection pressure during the transmission of moso bamboo indicated significant genetic differences among subpopulations. Furthermore, 3681 genes related to adaptability, stress resistance, photosynthesis, and hormone were identified from the selected regions. Four SNP markers developed and validated. Based on the population dynamics history and distribution simulation, we found that the distribution of moso bamboo has been influenced by the climate change in geological history.

**Discussion:**

These findings hold significant implications for enhancing our genetic comprehension of bamboo populations and exploring germplasm resources.

## 1 Introduction

Bamboo has made great contributions to the society, economy and ecology of China and the world, especially in the process of poverty reduction and rural modernization, because of its bioecological characteristics of one-time planting and sustainable utilization. With the deepening of bamboo research and the outstanding contribution of industrial development to the national economy and environmental protection, the host country of INBAR (International Network for Bamboo and Rattan, INBAR) was established in China in 1997. Moso bamboo (*Phyllostachys edulis*) belongs to the genus *Phyllostachys* of Gramineae, with a facultative clonal plant with long-term asexual reproduction, which is the largest number of natural widely distribution resources, the largest economic value and the most profound research in China (Song et al., 2020). According to the latest China Forest Resources Report, China’s bamboo forest area is 7.57 million hectares, and the current bamboo forest area accounts for 69.78% of the total bamboo planting area, reaching 5.28 million hectares. There are 13 provinces with distribution of moso bamboo forests in China, among which Fujian, Hunan, Jiangxi and Zhejiang have an area of more than 700,000 hectares, with a total area of 4.22 million hectares, accounting for 79.89% of the national moso bamboo forest area (China Forestry and Grassland Administration, 2021). Moso bamboo is tall, it is impossible to rely on long-distance and batch introduction of mother bamboo for afforestation in ancient times. The economic value of bamboo is extremely high, and it has a wide range of uses, including construction, furniture manufacturing, papermaking, and handicraft production. Due to its fast growth rate, early maturity and excellent material quality, it has an important economic position in agriculture, forestry and industry. The huge amount of bamboo resources, the wide geographical distribution and the strong economic industry make it an important pillar of China’s bamboo industry (Shi et al., 2020).

The growth of moso bamboo is closely related to its geographical location, and human activities and environmental changes have a certain impact on its natural population (Ahuja and Jain, 2016). A genome-scale investigation of the genetic diversity and population differentiation of moso bamboo across the entire natural distribution are essential for implementing appropriate conservation strategies to harness its biodiversity. In recent years, the advent of moso bamboo draft genome has ushered in a new epoch in its population genetics research (Peng et al., 2013; Zhao et al., 2018). Transcriptome studies, whole-genome resequencing (WGRS), and genome-wide association (GWAS) on trait candidate genes and pan-genome studies of moso bamboo have shown that the genetic diversity is low, the genotype heterozygosity is high, and its variation mainly exists between haplotypes. These studies provide important genomic resources for the protection and sustainable use of moso bamboo (Liu et al., 2020; Lan et al., 2021; Zhou et al., 2019; Zhao et al., 2021; Hou et al., 2024). The facultative clonal plants, which are widely distributed and have long-term asexual reproduction, have matured physiologically and have not seen large-scale flowering or sporadic flowering for a hundred years. The study of its subgroup structure has not been reported.

Studies on population genetics in other bamboos have also been reported, such as *Chimonobambusa tumidissinoda* J. R. Xue & T. P. Yi ex Ohrnb exhibited three distinct genetic groups and low levels of genetic diversity (Ye et al., 2024; Wang et al., 2024), while in natural populations of the Brazilian Amazon rainforest, *Guadua* species (Poaceae: Bambusoideae) were found to exhibit high average genetic diversity compared to other bamboo species (Silva et al., 2020). Low-depth sequencing confirmed ancient hybridization and allopolyploidy in the origin of the extant woody bamboo lineages (Chalopin et al., 2021), and genetic diversity, population structure, and gene flow analysis of various *Oxytenanthera abyssinica* (A. Rich.) Munro populations in Ethiopia demonstrated that genetic diversity is associated with distinct geographic regions (Abdie et al., 2020).

Recent studies on the genetic diversity of moso bamboo have been performed using various types of molecular markers. Among the Reduced-representation Genome sequencing (RRGS) techniques, genotyping by sequencing (GBS) emerges as a versatile, rapid and economical genomic method that combines marker discovery and genotyping. It has been extensively utilized to investigate genetic diversity and population structure of non-model species (Ji et al., 2022; Liu et al., 2021; Jia et al., 2020). Compared to traditional markers, GBS excels in detecting subtle changes in population structures, offering a more comprehensive description of genetic variation patterns in non-model species (Ji et al., 2022; Liu et al., 2021; Jia et al., 2020). Simplifying genome data proves instrumental in reshaping the evolutionary history of ancestors and enhancing our understanding of species formation and evolution (Parchman et al., 2018). For instance, the low genetic diversity structure of *Cryptomeria fortunei* was caused by climate fluctuation and genetic drift caused by human disturbance (Cai et al., 2020). The genomic data of *Primula tibetica* from Tibet show that it has multiple glacial refuges on the Qinghai-Tibet Plateau (Ren et al., 2017). The simplified genome population of *Circaeaster agrestis* unveiled abnormal and unbalanced genetic variation among the genetic differentiation was significantly changed by geographical and niche isolation (Zhang X. et al., 2020).

Species evolution is subject to diverse external pressures, resulting in various directions. Genome-wide data of cultivated and wild apples (*Malus domestica*) indicate that gene introgression may be an important driving force in the initial domestication of apples (Ma et al., 2017). According to the whole genome resequencing data of *Phaseolus vulgaris*, scholars have identified a group of genes related to increasing leaf mass and seed size (Schmutz et al., 2014). ZHANG and GUO constructed a phylogenetic framework of more than 200 bamboo species across more than 30 genera, including moso bamboo, and revealed the complex history of radiation evolution and reticulation evolution of bamboos by combining the diversity characteristics o and key trait evolution (Zhang Y. et al., 2020; Guo et al., 2019).

Climate change is widely acknowledged as a major threat to biodiversity. Multidisciplinary approaches that combine population genetics and species distribution modelling to evaluate these threats and recommend conservation actions are essential (Kimberly et al., 2013). Species Distribution Modeling (SDM) proves valuable for studying the impact of climate change on distribution, determining potential suitable areas, and mapping distribution areas. With the sharing of species distribution data and the rapid development of GIS technology, the simulation analysis of species geographical distribution has become an important application tool (Araújo and Luoto, 2007). MaxEnt uses existing distribution points and biological environment variables to estimate the ecological needs and potential distribution areas of species based on the maximum entropy principle (Connor et al., 2018; Elith et al., 2011). The under current and future climate scenarios of the rare bamboo species *Chimonobambusa tumidissinoda* potential distribution range predicted by MaxEnt model (Wang et al., 2024). The MaxEnt model was used to simulate the distribution pattern of *Ginkgo biloba* LIG (Last inter-glacial), LGM (Last Glacial Maximum) and contemporary (1970–2000 data) (Zhao et al., 2016).

Although moso bamboo holds immense economic and ecological value, there remains a scarcity of studies on the genetic variation of moso bamboo across its entire natural geographical distribution area. The population structure, population history and potential distribution of moso bamboo are not fully understood. In this study, we sequenced 193 individuals from 37 natural populations of moso bamboo and utilized genome-wide SNPs derived from GBS data to address the following questions (**Supplementary Figure S1**): (1) assessing the genetic diversity and population structure of moso bamboo, (2) developing and validating possible genetic loci related to the temperature stimulus of moso bamboo, (3) reconstructing the demographic history and potential distribution of moso bamboo. The single-nucleotide polymorphisms (SNPs) variations are further analyzed to understand genome features and population structure to aid in further research and applications. The identified variations provide insight into the origin and evolutionary history of moso bamboo. These findings will contribute to a comprehensive understanding of the evolutionary history of moso bamboo, providing a molecular foundation for genetic rescue efforts in its natural populations and enhancing the scientific and technological capabilities in collecting, evaluating, preserving and utilizing moso bamboo germplasm resources.

## 2 Materials and methods

### 2.1 Sample collection

A total of 193 leaf samples of *Phyllostachys edulis* were collected from 37 localities in natural distribution of China during several filed campaigns (**Supplementary Table S1**, **Supplementary Figure S2**). In general, more than five individuals per population were sampled, while the following exceptions: populations No. 7, 13, 27, and 29 (three populations each), No. 14 and 19 (four populations each), and No. 31 and 32 with only two individuals, respectively. The minimally sample size for populations No. 31 and 32 from Sichuan Yibin reflects the challenging field conditions in this region, where low natural regeneration rates of moso bamboo, wildfire and steep terrain significantly limited natural populations sample accessibility. The sampled young leaves were directly silica dried and voucher samples were deposited at Nanjing Forestry university.

### 2.2 DNA extraction, library preparation and sequencing

Total genomic DNA of 193 moso bamboo samples from 37 populations was extracted from dried leaves using the improved CATB method (Doyel et al., 1996). Subsequently, the DNA purity and concentration were checked by the NanoDrop 1000 spectrophotometer (NanoDrop Technologies, Wilmington, DE, USA) and assessed using agarose gel electrophoresis with a concentration of 1.0%. The GBS library was constructed following the protocol developed by Qi et al. (2018), with a selected size range of 300–600 bp. Briefly, cleaned DNA was digested using 8 U MspI and 8U PstI-HF (NEB), followed by T4-ligase ligation, random shearing and size selection. The recovered DNA fragments were used as templates for PCR amplification. After purification and quality checking, the resulting library was sequenced with paired-end (2×150 bp) reads on the Illumina Novaseq PE150 platform (Illumina, San Diego, USA) at Beijing Kaitai Gene Technology Co., Ltd. (Beijing, China).

All of the raw sequencing data have been submitted to the NCBI Short Reads Archive database with the accession number PRJNA1133081, ensuring that the data are publicly accessible and reproducible.

### 2.3 SNP and InDel calling

Raw reads were filtered using fastp (version 0.23.1) to obtain clean reads (Chen et al., 2018). Data quality filtering was performed in three sequential steps: first, reads containing adapters were removed; second, reads containing more than 10% of ambiguous bases (N) were discarded; and third, low-quality reads were eliminated, defined as those in which the number of bases with a quality score (Q) ≤ 10 exceeded 50% of the total read length. Clean reads were then aligned to the previously assembled genome of *P. edulis* using BWA (version 0.7.8) (Li and Durbin, 2010). Subsequently, based on the comparison results, the Haplotypecaller module of GATK software (version 4.1.8) was employed for SNP and InDel detection (Mckenna et al., 2010). The raw SNP and InDel dataset were further filtered by vcftools software (concrete parameters: ‘–maf 0.01 –max-missing 0.8 –minDP 4 –min-alleles 2 –max-alleles 2’, version 0.1.16) (Danecek et al., 2011). The filtering criteria were applied as follows: reads were retained for sites with a minimum depth of 4; loci with a minor allele frequency (MAF) less than 0.01 were removed; and loci with SNP genotyping deletion rate exceeding 20% were excluded.

### 2.4 Population genetic analysis

Population genetic analysis was conducted based on the filtered high-quality SNP data. Phylogenetic tree among individuals was constructed using the maximum likelihood (ML) algorithm of Evolview (http://www.evolgenius.info/evolview). The population structure was analyzed using Admixture (version 1.3.0) (Porras-Hurtado et al., 2013), with the assumed number of subgroups (K) ranging from 2 to 10. The optimal K value determined based on the minimum cross-validation (CV) error. Principal component analysis (PCA) and kinship analysis among individuals were performed using gcta (version 1.26.0) (Yang et al., 2011).

Genetic diversity indicators, including Hardy Weinberg equilibrium (HW-P), Number of alleles (*Na*), effective numbers of alleles (*Ne*), observed heterozygosity (*Ho*), expected heterozygosity (*He*), polymorphism information content (PIC), nucleotide diversity (π), fixation index (Fst) and inbreeding coefficient (Fis, Fit) were calculated by Genepop (version 4.7) in R (version 4.2.3) (Rousset, 2008). The Reynolds genetic distance between populations (DR) was calculated by Fst, DR=−ln(1−Fst) .

Mantel tests were performed to evaluate the associations between genetic distance, geographic distance and environmental distance with 9999 permutations in the R package vegan. AMOVA analysis was carried out by Arlequin (version 3.5.2.2) to quantify the genetic variation among and within the genetic groups of moso bamboo inferred by the population structure analyses. DnaSP (version 6.0) was used to calculate the gene flow (Nm) of moso bamboo population (Rozas et al., 2017).

Functional annotation of all candidate genes was performed using EGGNOG-Mapper (http://eggnog-mapper.embl.de/). Gene Ontology (GO) and Kyoto Encyclopedia of Genes and Genomes (KEGG) enrichment analysis was performed on the annotation results using TBtools (version 1.9) (Chen et al., 2020).

### 2.5 Validation of SNPs

To validate SNPs identified from GO and KEGG enrichment, three of nonsynonymous mutation sites and one of synonymous site in the coding region of functional genes related to temperature stimulation response were selected for PCR amplification and Sanger sequencing. Flanking sequences of selected SNPs were extracted from the reference genome and PCR primers were designed with Premier Primer 5 (Singh et al., 1998) and synthesized by Beijing Tsingke Biotech Co., Ltd (Beijing, China) (**Supplementary Table S2**). DNA samples of ten individuals from each of the three subpopulations were randomly selected as templates for PCR amplification. PCR reaction system: 1.1×T3 Super PCR Mix 22μL, 10μM Primer F 1μL, 10μM Primer R 1μL, Template (gDNA) 1μL. PCR reaction conditions: 98°C 2 min; 98°C 10 s, 60°C 30 s, 72°C 45 s, 35 cycles; stretched at 72°C for 5 min and stored at 4°C. The amplified PCR products were sequenced by the Sanger method and sequencing results were analyzed with BioEdit (Hall, 1999).

### 2.6 Demographic history analysis

The Tajima’s D and Fu ‘s Fs values of moso bamboo were calculated by Arlequin (version 3.5) (Fu and Li, 1993). The site frequency spectrum (SFS) of each subgroup was calculated by the easySFS.py script (https://github.com/isaacovercast/easySFS). Stairway Plot (version 2.1) was used to infer the demographic history of moso bamboo (Liu and Fu, 2020).

Species data was obtained from the Global Biodiversity Information Facility (GBIF, https://www.gbif.org) and 19 bioclimatic factors (**Supplementary Table S3**) with a spatial resolution of 2.5 arc-minutes (~ 5 km) were downloaded from the PaleoClim (http://www.paleoclim.org/) database. Using ArcGIS (version 10.5), SDMtoolbox (version 2.10.4) and MaxEnt (version 3.4.2), the geographical distribution of moso bamboo was simulated and analyzed during three geohistorical history periods, the last interglacial (LIG) 130000–116000 years ago, the last glacial maximum (LGM) 21000 years ago and the current period (Ct, 1979–2013) (Steven et al., 2017).

## 3 Results

### 3.1 Analysis of GBS data and SNP discovery

**Figure 1:**
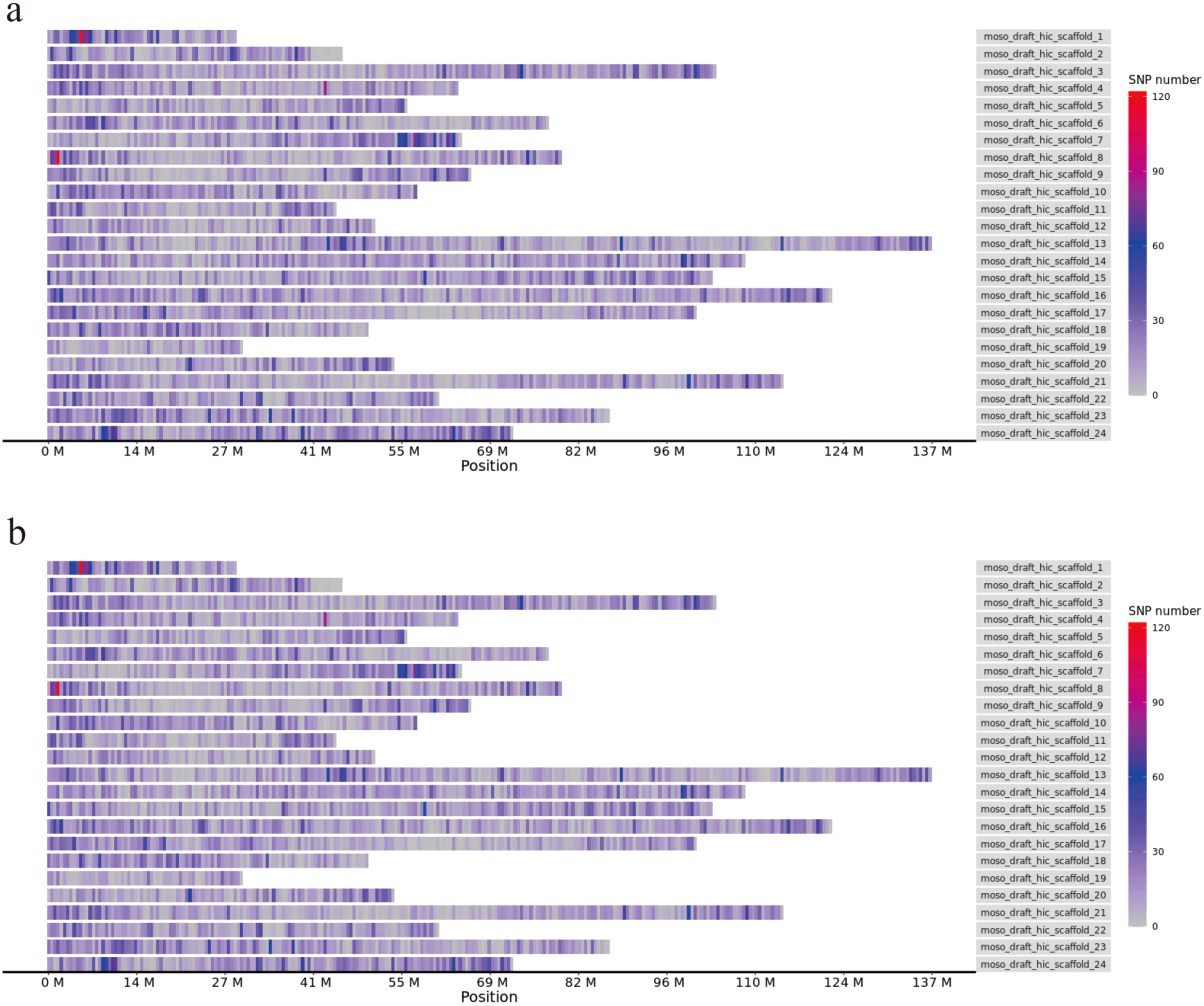
Chromosome distribution of SNPs, InDels. **(a)** SNP labeling density. **(b)** InDel labeling density.

A total of 1,824 million reads, 248.16 GB clean data was obtained from 193 moso bamboo by GBS (**Supplementary Table S4**). The data size in 193 moso bamboo samples ranged from 0.90 to 2.43 GB. The clean data was aligned to the moso bamboo genome, the mapping rates were raged from 34.93% to 99.09%, with a mean alignment rate of 89.18% and an effective sequencing depth of 11.65×. Using our specified parameter settings, we identified 51,033 raw SNPs and 7,664 InDels across the 193 samples using bcftools (**Figures 1a, b**). The distribution of SNPs on 24 chromosomes of moso bamboo was examined, and the density of SNP markers was calculated. It was found that there was one SNP variant site per 3518 bp, covering the whole genome of moso bamboo. the density of SNP markers at the beginning and end of each chromosome was higher than that in the middle (**Figure 1a**). Through the statistical analysis of the SNPs annotation results, it was found that the SNPs were located in the Intergenic region (**Supplementary Table S5**).

### 3.2 Population genetic structure

**Figure 2:**
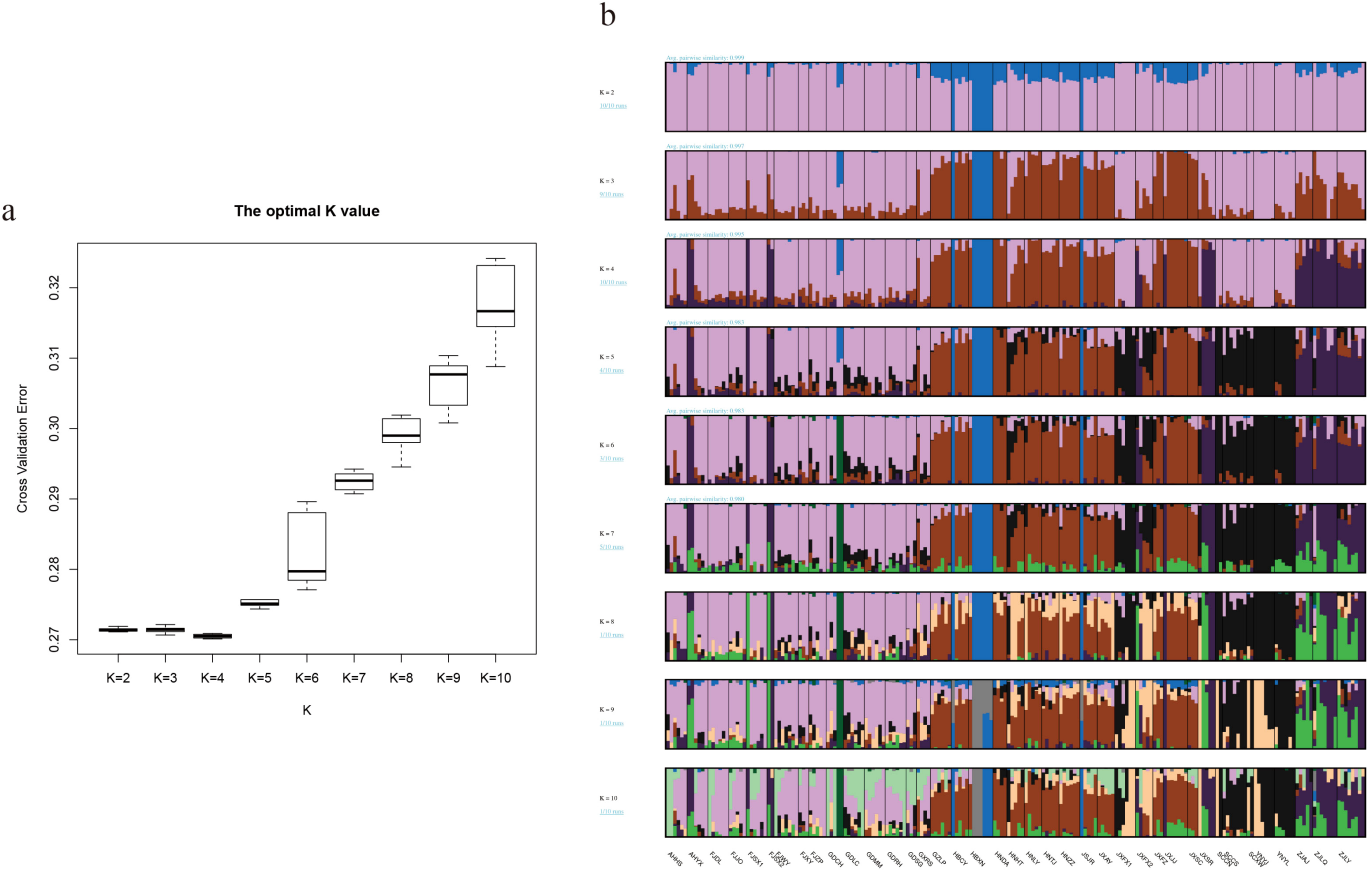
Analysis of population structure of moso bamboo. **(a)** Cross validation (CV) error value for different model (K) **(b)** Subpopulation structure of moso bamboo. The x-axis shows moso bamboo populations, and the y-axis quantifies the proportion of inferred ancestral lineages.

ADMIXTURE was used to analyze the genetic structure of the 193 samples collected from the whole natural distribution area. The number of subsets (K) was set from 2 to 10, repeat 10 runs and perform ten-fold cross validation. The optimal number of subsets was determined based on the K value corresponding to the minimum CV (Cross Validation) error. It was found that K = 4, with a CV value of 0.270, was the most appropriate number according to the CV error (**Figure 2a**). This suggests the presence of four potential subpopulations in the entire Chinese moso bamboo population. Unsupervised clustering analysis revealed that distinct subgroups such as the Hunan-Jiangxi subclass, the lower Yangtze River subclass, the southeast coast, the Zhujiang River Basin subclass in South China, and isolated samples in Xian ‘an District, Xianning City, Hubei Province. Some individual samples were found alongside others, reflecting the natural genetic structure traits of moso bamboo (**Figure 2b**).

**Figure 3:**
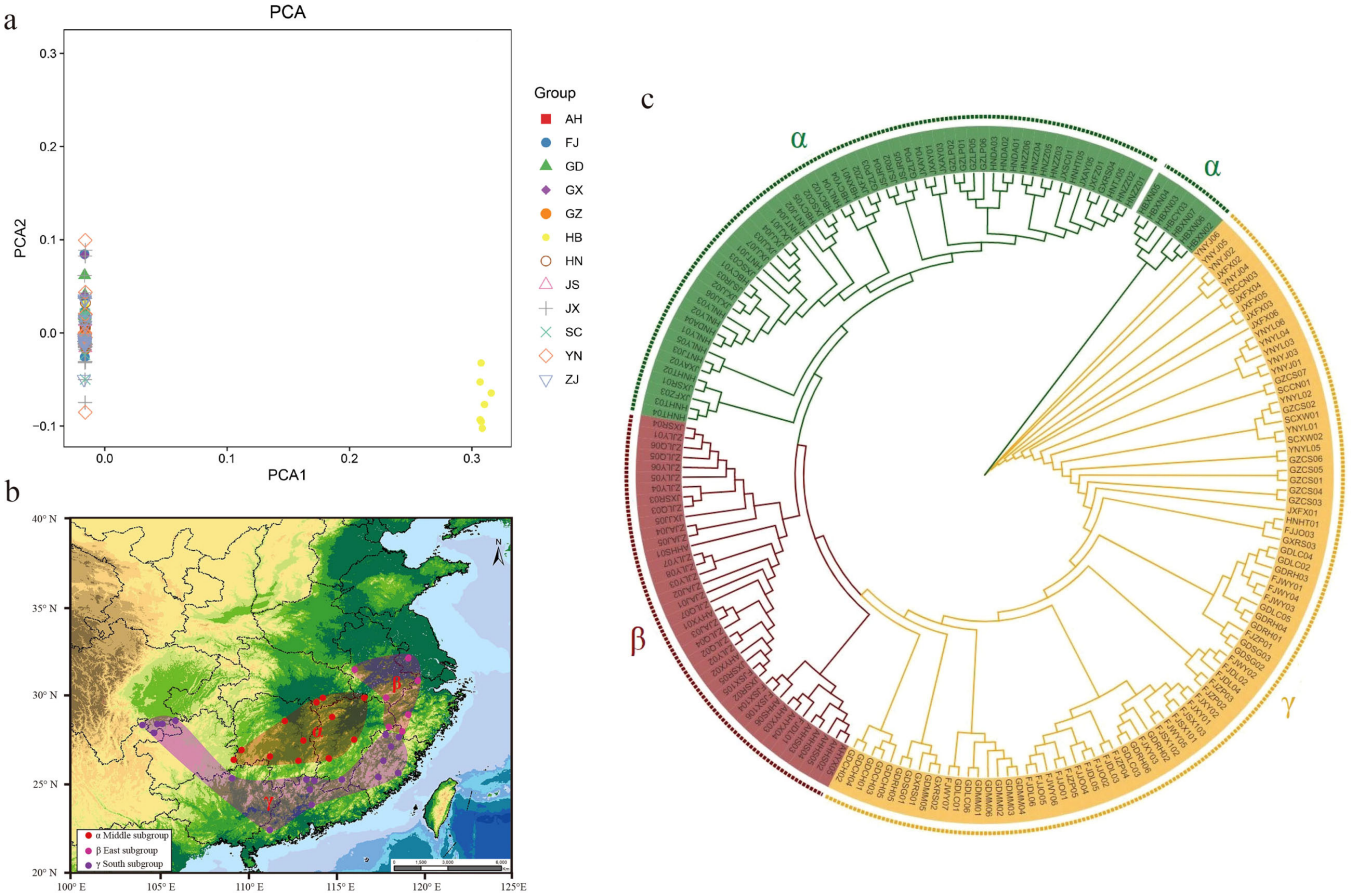
Three subpopulation structure of moso bamboo. **(a)** PCA analysis of moso bamboo, the PCA1 was 96.37% and the PCA2 was 3.63%. **(b)** Moso bamboo subpopulation distribution of China. Dots represent sampling distribution points, and colors represents subpopulations. Red shows central alpha subpopulation, pink shows eastern beta subpopulation, and purple shows southern gamma subpopulation. **(c)** Phylogeny tree of moso bamboo based on neighbor-joining method. Green represents central α-subpopulation, red represents eastern β-subpopulation, and orange represents southern γ-subpopulation.

To better understand the population structure of moso bamboo, PCA and phylogenetic tree were performed. Principal components PC1 and PC2 accounted for 52.91% and 26.77%of the variance, respectively (**Supplementary Table S6**). The results indicated that moso bamboo was divided into two subpopulations, with 186 samples clustered on the left (96.37%), while only 7 samples from Xian ‘an (6) and Chongyang (1) in Hubei Province gathered on the right (**Figure 3a**). The phylogenetic tree of 193 moso bamboo divided into three genetic groups (**Figure 3b**). Considering China ‘s topography, the large mountains significantly affected the natural distribution of moso bamboo. Consequently, the natural distribution of moso bamboo in China was divided into three potential subpopulation structures (**Figure 3c**). The central α-subpopulation, known as the Luoxiao Mountain group, located in the middle reaches of the Yangtze River catchment area between Luoxiao-Xuefeng Mountains at the junction of Jiangxi and Hunan, north to Lushan City, Jiangxi, south to Leiyang and Dong ‘an, Hunan, mainly distributed in Mufu Mountain, Jiuling Mountain, and Wugong Mountain regions. The eastern β-subpopulation, known as the lower reaches of the Yangtze River population, located below Hukou, Jiangxi. The samples include Huoshan, Yixian, Jurong, Anji, Longyou, Longquan, Shangrao, and the population samples are almost clustered in one branch. The southern γ-subpopulation, known as the Zhujiang River Basin, located from the basins along the southeast coast of China to the Zhujiang River Estuary, extending westward to the Sanchahe area of the Xijiang River Basin.

### 3.3 Genetic diversity and origin of population variation

Based on the SNPs set constructed by 37 moso bamboo populations, we conducted an analysis of their genetic diversity. The results indicated that at the species level, the HW-P (Hardy-Weinberg) value was between 0.638 and 1.000, suggesting that all moso bamboo reached the Hardy-Weinberg genetic balance (*P* > 0.05), with the balanced gene and genotype frequencies in each population (**Supplementary Table S7**). At the population level, the average observed number of alleles (*Na*) was 1.270, the effective number of alleles (*Ne*) was 1.210, the expected heterozygosity (*He*) was 0.113, the observed heterozygosity (*Ho*) was 0.182, and the polymorphism information content (PIC) was 0.087. Notably, the observed *Ho* values in moso bamboo populations in Hubei tended to be relatively high, while those in Guangdong seem to be generally low. The nucleotide diversity (π) of moso bamboo ranged from 0.105 to 0.280, with an average of 0.129.

**Figure 4:**
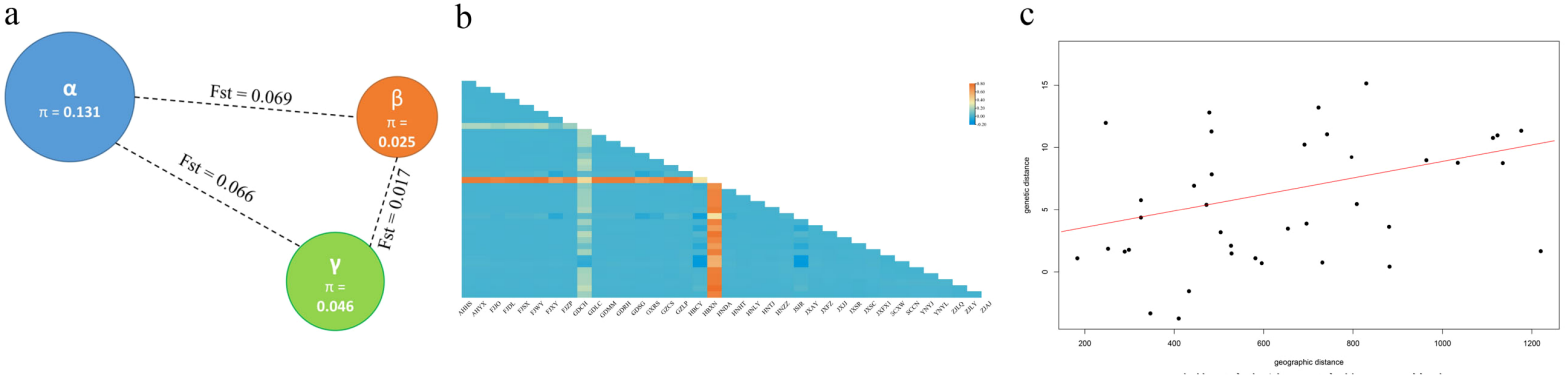
Genetic variation of moso bamboo population. **(a)** Diversity (π) and the genetic distance (Fst) among subpopulations. The color represents sample subpopulation, blue represents central α-subpopulation, orange represents eastern β-subpopulation, and green represents southern γ-subpopulation. The radius of pie represents genetic diversity, and dashed line length represents Fst value between two subpopulations. **(b)** Genetic distance matrix of moso bamboo populations. **(c)** Results of the Mantel test of the relationship between geographic distance and genetic distance.

In order to explore the degree of genetic differentiation among moso bamboo subpopulations, the F-statistics (Fis, Fst and Fit) were calculated and evaluated for the population differentiation index among each subpopulation (**Figure 4a**). According to Robert ‘s theory (Robert and Samuel, 1978), the value of genetic differentiation coefficient distinguished three levels, 0< Fst< 0.05 (low differentiation), 0.05< Fst< 0.25 (moderate differentiation) and 0.25< Fst (high differentiation). The analysis revealed that the Fst values among the three subpopulations were all above 0.05, indicating a moderate level of genetic differentiation among the moso bamboo subpopulations. Specifically, the Fst values of the central α-subpopulation were higher compared to those of the eastern β-subpopulation and southern γ-subpopulation, indicating that the genomes of the eastern and southern populations were closer, suggesting a higher frequency of gene exchange between them, yet distinct from the central population.

The genetic and geographic distance matrix among 37 populations of moso bamboo indicated that genetic distance ranged from -0.136 to 0.798, with an average of 0.042 (**Figure 4b**). Mantel tests showed a non-significant correlation between genetic and geographic distances for the 37 moso bamboo populations (*r* = 0.1664, *p* = 0.7511; **Figure 4c**). These results indicate varying different degrees of genetic variation among populations, with an overall small genetic variation.

AMOVA analysis demonstrated that 93.86% of the total variation was within populations, while only 6.14% was observed among groups (**Table 1**). The gene flow (*Nm*) was less than 1.0, indicating that the genetic variation of moso bamboo population primarily originated from within the groups.

**Table 1:** SNP AMOVA on moso bamboo populations.

Source of variation	*df*	Sum of squares	Variance components	Percentage of variation	Fst	Nm
Inter populations	2	2846.862	18.806 Va	6.14	/	/
Within populations	190	54624.133	287.495 Vb	93.86	/	/
Total	192	57470.995	306.301	100	0.081	3.210

### 3.4 Selection pressure of moso bamboo population

**Figure 5:**
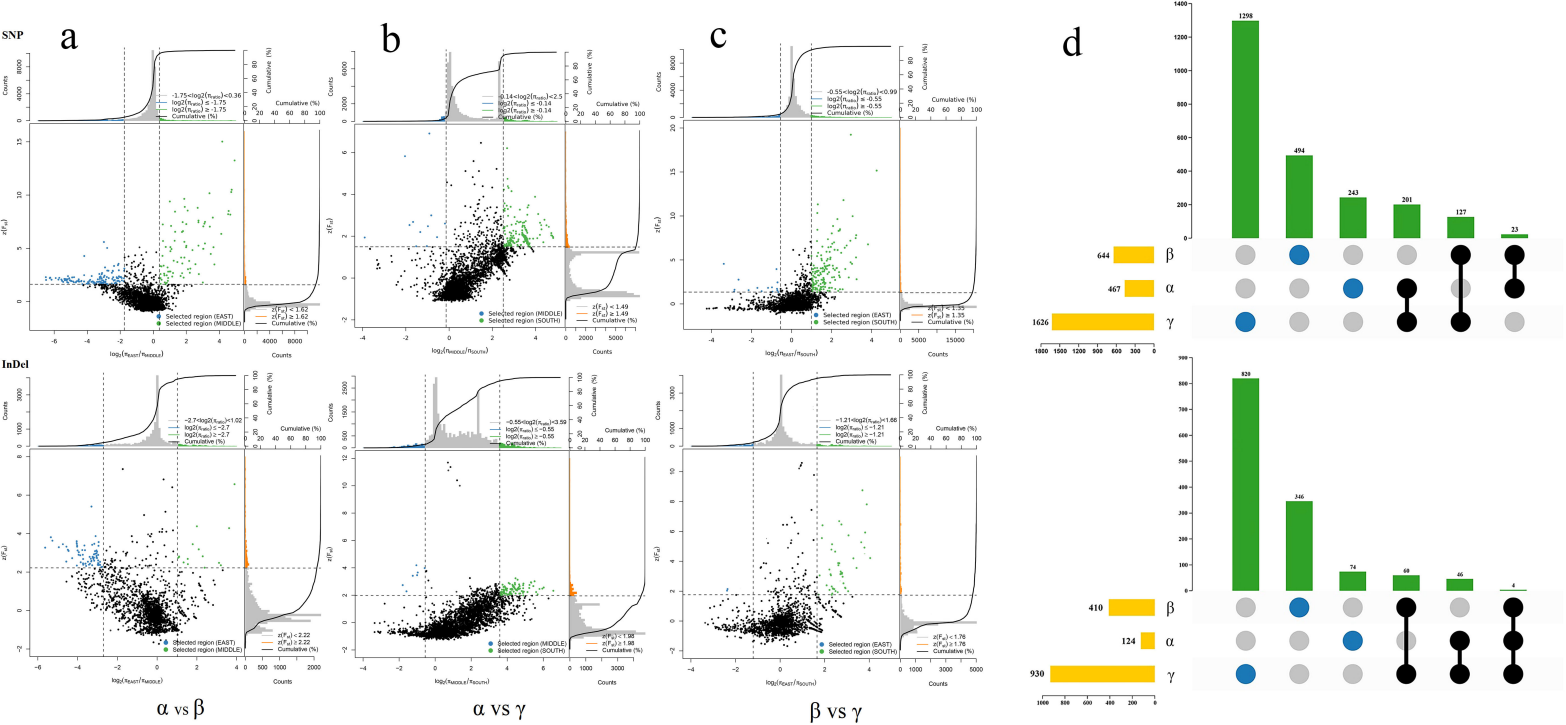
π ratio, Fst value distribution and selection pressure of moso bamboo subpopulation. **(a)** α vs β. **(b)** α vs γ. **(c)** β vs γ. The data points are located to the left and right of the vertical dotted lines (corresponding to the 5% left and right tails of the log_2_(π ratio) distribution, -1.75 and 0.36, -0.14 and 2.50, -0.55 and 0.36, -2.70 and 1.02, -0.55 and 3.59, -1.21 and 1.66, respectively). Above the horizontal dotted line (5% Fst right tail of the distribution, with Fst of 1.62, 1.35, 1.49, 2.22, 1.98, 1.76) was identified as the selection area for the two subpopulations (blue and green dots), respectively. X-axis represents enrichment factor, y-axis represents pathway name, dots size represents number of genes and dots color represents p-value. **(d)** Statistical map of selected genes of bamboo subpopulations. Yellow bars represent total number of genes, green bars represent unique or shared genes, blue circles represent unique genes, and black connecting circles represent shared genes.

To investigate the selection pressure on moso bamboo during transmission, we used the constructed SNPs and InDels to calculate the nucleic acid diversity (π) ratio and Fst value within100 kb sliding window of the genome between different groups. We identified 693 windows based on geographical differentiation, selecting regions with significantly low or high π values in the top 5% and high Fst values in the top 5% as the windows of interest (**Figures 5a-c**). These selected regions covered a total of 3681 genes as determined by aligning these windows to the new reference genome annotation file of moso bamboo (**Figure 5d**). Each subpopulations experienced distinct selection pressures, with a significant proportion of unique genes in each, showing obvious differences in the selected genes (**Figure 5d**; **Supplementary Table S8**).

**Figure 6:**
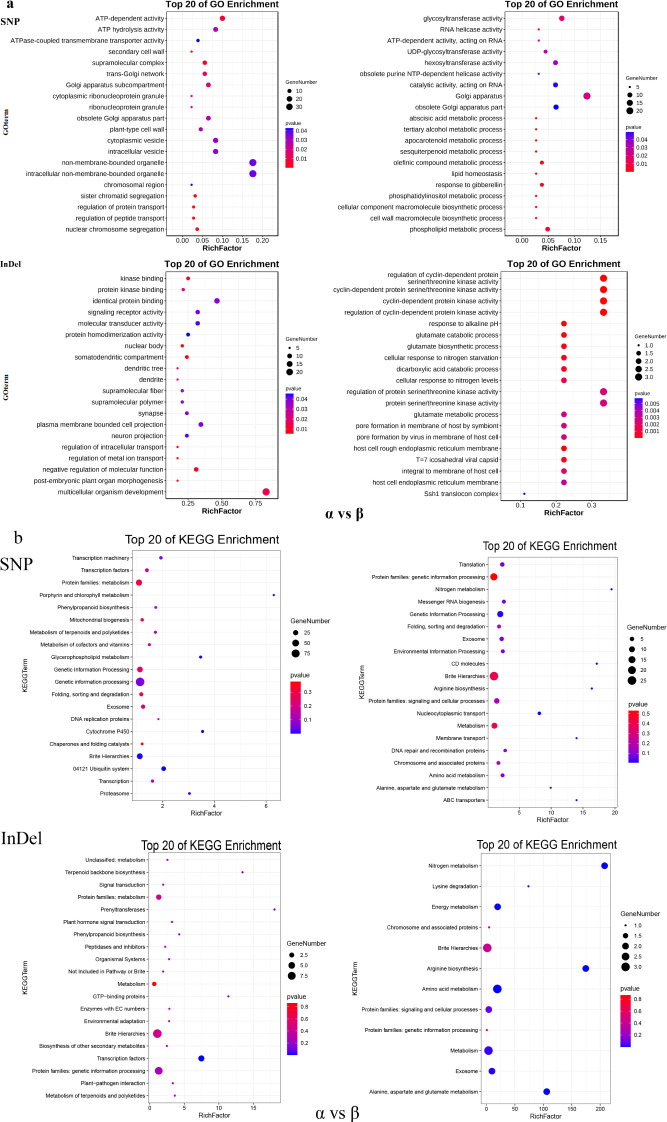
GO and KEGG analysis of selected genes of moso bamboo subpopulation. **(a)** GO analysis of elected genes between α-subpopulation and β-subpopulation. **(b)** KEGG analysis of elected genes between α-subpopulation and β-subpopulation. X-axis represents rich factor, y-axis represents pathway name, dots size represents gene number, and dots color represents p-value, Vacancies indicate no selection genes and no GO and KEGG analysis results.

In order to explore the genes and their functions corresponding to the selection pressure of moso bamboo, GO and KEGG enrichment analysis revealed distinct gene enrichments among the three subpopulations (**Figure 6**). In the central α-subpopulation, significant enrichment in processes such as abscisic acid metabolism, carotenoid metabolism, olefin compound metabolism, response to gibberellin, phospholipid metabolism, cell wall macromolecular biosynthesis, transmembrane transport and transmembrane transporter activity, organelles and plasma membrane, macromolecular catabolism, and endoplasmic reticulum related genes (**Figure 6a**; **Supplementary Figure S3**). The significant KEGG enrichment in processes such as genetic information, nucleocytoplasmic transport, nitrogen metabolism, arginine biosynthesis, membrane transport, alanine, aspartate and glutamate metabolism, amino acid metabolism, energy metabolism and mitochondrial biogenesis related genes (**Figure 6b**; **Supplementary Figure S4**). The eastern β-population exhibited significant enrichment in genes related to supramolecular complexes, regulation of protein transport, temperature stimulation response, abscisic acid biosynthesis and regulation, and carotenoid biosynthesis (**Figure 6a**; **Supplementary Figure S5**). The significant KEGG enrichment in genes related to brite Hierarchies, ubiquitin system, glycerophospholipid metabolism, porphyrin and chlorophyll metabolism, mitochondrial biogenesis, and transcription factors (**Figure 6b**; **Supplementary Figure S6**). In the southern γ-subpopulation, enrichment was observed in catalytic activity, RNA polymerase II transcription regulatory complex, secondary cell wall, cell surface receptor signaling pathway, cell response to endogenous stimuli, hydrolase activity genes, stimulus response-related genes, secondary cell wall genes, defense response to bacteria, and chloroplasts and chloroplast envelopes (**Supplementary Figure S3**; **Supplementary Figure S5**). Significant KEGG enrichment was observed in genetic information, replication and repair, nucleocytoplasmic transport, amino sugar and nucleotide sugar metabolism, lipid biosynthesis proteins, circadian rhythm plant, fatty acid biosynthesis, nitrogen metabolism, arginine biosynthesis alanine, aspartate and glutamate metabolism and energy metabolism (**Supplementary Figure S4**; **Supplementary Figure S6**).

By screening the SNP difference sites of the functional genes related to temperature stimulation response in the three subpopulations, the SNPs with stable polymorphism differences were selected, and a total of four SNP sites with good typing were screened. Finally, two nonsynonymous mutation sites (SNP: Chr24_67953493, Chr24_67953514) and one synonymous mutation site (SNP: Chr24_67953497) were screened out in *PH02Gene00430* gene, and one nonsynonymous mutation site (SNP: Chr1770_920) was screened out in *PH02Gene21923* gene (**Supplementary Table S9**). Ten samples from each of the three subpopulations were randomly selected as DNA templates for PCR amplification, and the amplified products were subjected to Sanger sequencing. The results showed that HBXN and HBCY in α subpopulation and JSJR in β subpopulation were polymorphic (**Supplementary Table S9**, **Supplementary Figure S7**). The primers of three nonsynonymous SNP mutations can be used to quickly detect the ability of bamboo temperature stimulation response, which are valuable SNPs molecular marker.

### 3.5 Demographic history and potential distribution of moso bamboo

**Figure 7:**
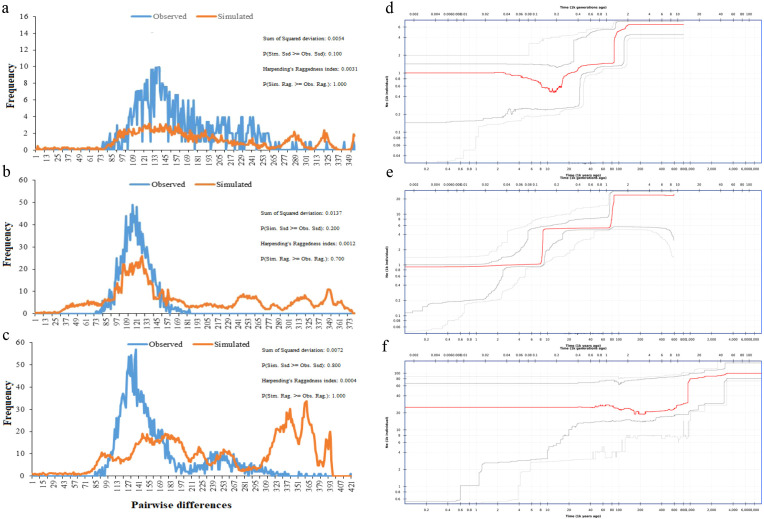
Nucleotide mismatch distribution and Population dynamics history of moso bamboo subpopulations. **(a, d)** α-subpopulation. **(b, e)** β-subpopulation. **(c, f)** γ-subpopulation. Red line represents median of 200 inferences based on subsampling. Dark gray lines represent 75% confidence interval of the inference. Light gray lines represent 95% confidence interval of the inference.

According to the neutral test results of moso bamboo test materials (**Table 2**), the Tajima ‘D and Fu ‘s Fs values for the whole population of moso bamboo, as well as for the central α-subpopulation and the eastern β-subpopulation, were negative but not statistically significant (*p >* 0.05) (**Figures 7a-c**). This indicates the absence of significant population expansion events in the history of Chinese moso bamboo across the population, as well as the α-subpopulation and the β-subpopulation. However, the test D and FS values of the southern γ-subpopulation were negative, with a significant p-value. The mismatch distribution analysis did not support the sudden expansion of the population (*r* = 0.0004, *p* > 0.05) (**Figures 7d-f**). These results show that the moso bamboo population and its subgroups have remained relatively stable state through their historical evolution, without experiencing obvious population expansion events. The historical population dynamics of moso bamboo showed that there were population bottlenecks effect about 8–30 ka (thousand years) and 70–120 ka years ago (**Figures 7d-f**). This stability is related to the growth habit of moso bamboo, characterized by its scattered bamboo nature and reliance on underground stems for rhizome growth every year.

**Table 2:** Neutrality tests of moso bamboo population.

Neutrality tests	Statistics	population	α-subpopulation	β-subpopulation	γ-subpopulation
Tajima’s D test	Tajima’s D	-2.206	-1.613	-0.755	-2.562
	P-value	0.013^*^	0.023^*^	0.244	0^**^
Fu’s FS test	FS	-0.700	-3.408	-0.683	-7.581
	P-value	0.114	0.073	0.205	0.012^*^

* indicates P < 0.05, ** indicates P < 0.01.

**Figure 8:**
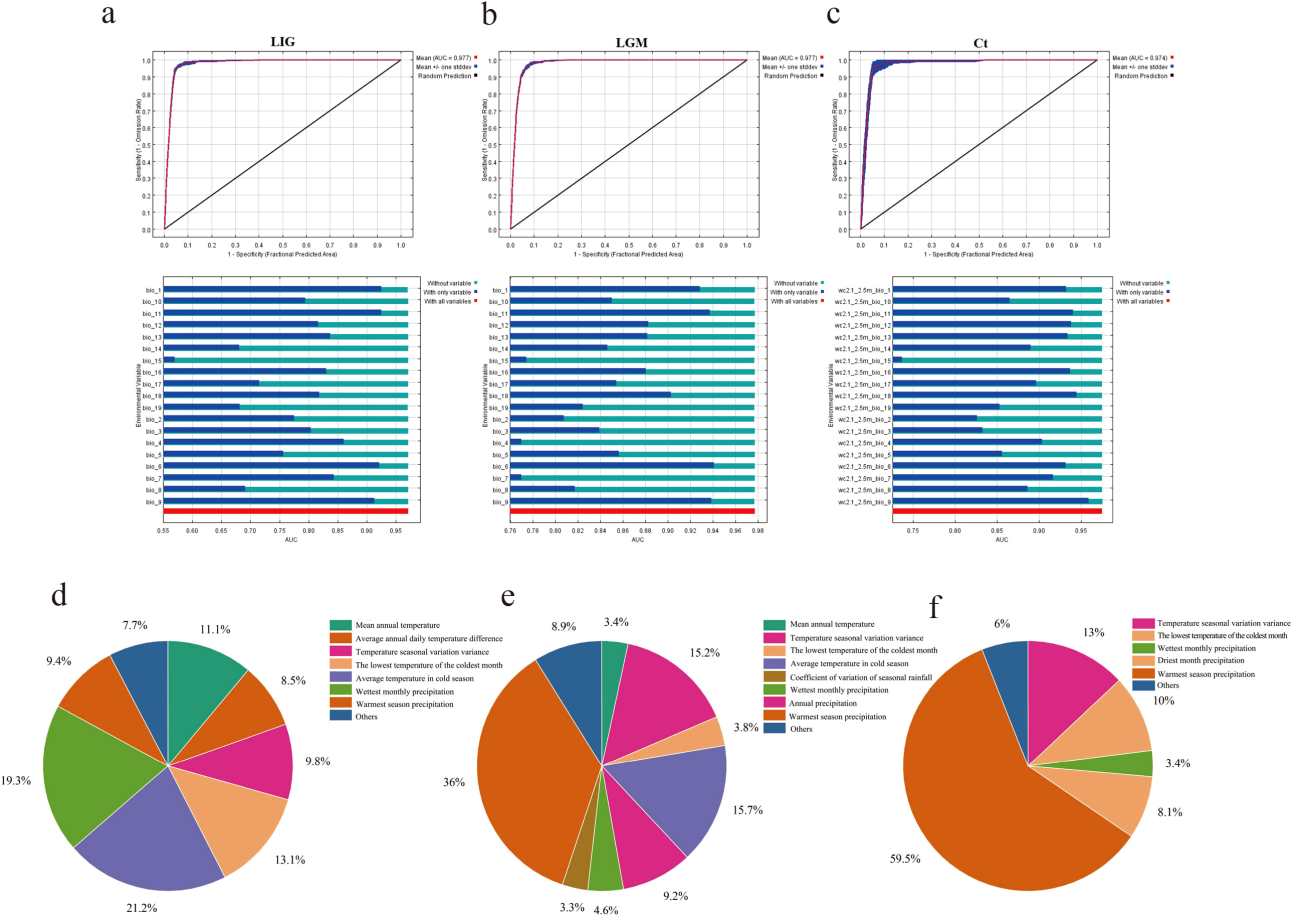
The geological history of moso bamboo simulated by MaxEnt. **(a-c)** ROC curve and jackknife test for forecasting three periods of moso bamboo based on MaxEnt model. **(d-f)** The contribution rate of climatic factors in the geological history of moso bamboo simulated by MaxEnt. **(d)** LIG period. **(e)** LGM period. **(f)** The current period.

In order to explore the adaptive distribution of moso bamboo in China, we meticulously selected distribution points with intervals exceeding 10 km, and finally obtained 342 valid distribution points. We then selected 19 bioclimates from WorldClim (**Supplementary Table S3**) for MaxEnt modeling. Our approach involved utilizing 75% of the moso bamboo distribution site data for training modeling, conducting 10 repeated runs, and using the remaining 25% of the distribution site data as a validation subset. The model’s accuracy was assessed based on the highest value of the receiver operating characteristic (ROC) in the Area Under ROC Curve (AUC), providing insight into the predicted geographical distribution areas during three historical geological and climatic periods, the last interglacial period (LIG) 130 000-116–000 years ago (**Figure 8a**), the last glacial period (LGM) 21–000 years ago (**Figure 8b**), and the current period (Ct) 1979-2013 (**Figure 8c**). Across these periods, the model exhibited high accuracy, with average AUC values of 0.977 for both LIG and LGM, and 0.974 for the current period, respectively, indicating robust predictive performance.

**Figure 9:**
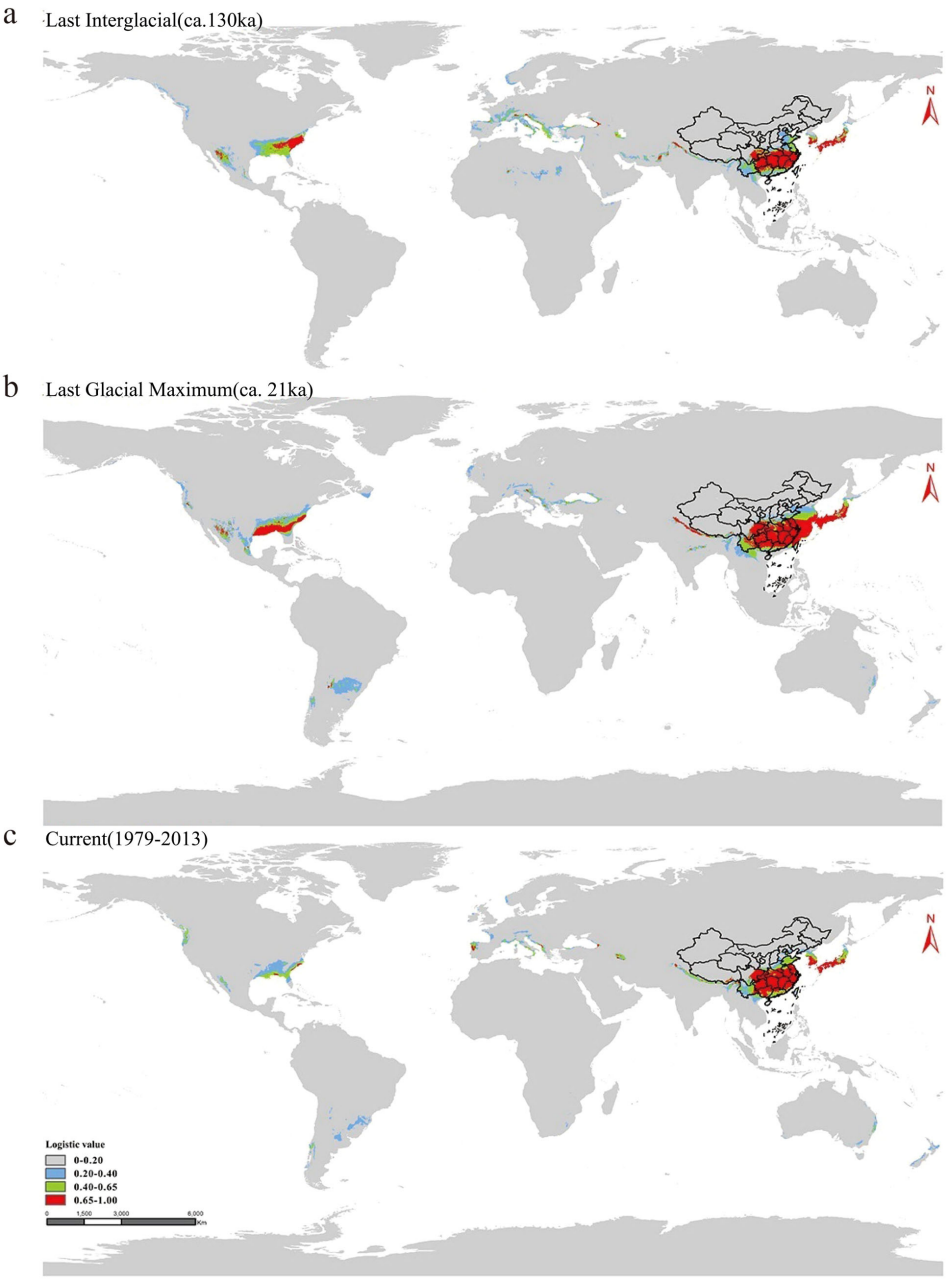
Distribution area of moso bamboo in geological history. **(a)** The last interglacial period (LIG). **(b)** The last glacial period (LGM). **(c)** The current period (Ct). Color indicates probability of suitable habitat for moso bamboo.

Based on the contribution of bioclimatic variables during the three periods, the five predominant factors influencing the prediction potential distribution area of moso bamboo were the warmest season precipitation, temperature seasonality, wettest monthly precipitation, the lowest temperature of the coldest month and annual mean temperature (**Figures 8d-f**). Notably, the warmest season precipitation factor had the most substantial impact. These five key factors effectively analyze the modern natural distribution results of moso bamboo. According to the value of LV (Logistic value), the moso bamboo habitat was divided into four grades. The core suitable area with LV 0.65-1.00 (**Figure 9a**), the middle suitable area with LV 0.40-0.65 (**Figure 9b**), the low suitable area with LV 0.20-0.40, and the non-suitable area with LV 0-0.20 (**Figure 9c**). Throughout the three historical periods, the simulated distribution area of moso bamboo globally exhibited a shrinking trend, with a significant reduction in the core suitable area.

## 4 Discussion

### 4.1 The population origin of natural distribution area of moso bamboo

Due to the natural barriers such as mountains and rivers, historical human activities have affected the gene flow among moso bamboo populations, resulting in genetic differentiation and the development of distinct subpopulations structures. The origin of moso bamboo has limited studies because of unsuitability for preservation and lack of fossil evidence. In this study, employing PCA and phylogenetic tree analysis, combined with niche distribution, the Chinese population of moso bamboo divided into three subgroups, central α, eastern β and southern γ. Notably, populations of moso bamboo in Yunnan, Guizhou and Sichuan were found to belong to the same subgroup as those in Fujian. The central α-subpopulation exhibited the highest nucleotide diversity, indicating that it serves as a focal point for variation and diversity. According to the theory of the center of origin of cultivated plants (Hummer and Hancock, 2015), the α-subpopulation in central China may be the origin center of moso bamboo, which is consistent with the results of this study. Additionally, research utilizing SSR and SNP markers has further confirmed this result (Zhao et al., 2021; Hou et al., 2024; Jiang et al., 2017).

### 4.2 Genetic diversity of natural populations of moso bamboo

The genetic diversity indexes of 37 populations in China were subjected to statistical analysis. The results of *He*, *Ho*, *Na*, *Ne*, Fis and Fit at the population level of moso bamboo showed a low level of genetic variation and excess of heterozygotes within the domestic moso bamboo population. This is similar to the results of genetic diversity and genetic structure analysis of 34 populations of moso bamboo in China by SSR technology (Jiang et al., 2017).

It is well understood that genetic drift, historical bottlenecks, and climate fluctuations (such as glacial and interglacial cycles) can significantly impact population size, leading to reduced genetic diversity. These factors are particularly relevant in long-term asexual reproductive plants. The evolution of both metapopulations and stable populations is primarily driven by genetic drift and genetic bottlenecks, resulting in subpopulations with low genetic diversity (Angst et al., 2022). In our study, the genetic diversity of moso bamboo population is notably low, while heterozygosity is high, affected by genetic drift, genetic bottlenecks (8–30 ka and 70–120 ka years ago), climate change, human factors and the reproduction characteristics of moso bamboo. Moso bamboo undergoes prolonged vegetative reproduction stages and the infrequent flowering intervals, yet its flowering and seed dispersal exhibit distinct characteristics. At the subgroup level, it was observed that the middle α-subpopulation displayed the highest genetic diversity. Furthermore, the genetic differentiation between the eastern β-subpopulation and southern γ-subpopulation was relatively minor, suggesting that more frequent gene exchange between them. This is consistent with the results of resequencing of moso bamboo populations in 15 representative regions of China (Zhao et al., 2021; Hou et al., 2024).

The AMOVA analysis revealed that the genetic variation within populations exceeded that among populations, a common trend in outcrossing and asexually reproducing perennial species. These species typically exhibit high levels of heterozygosity and maintain significant genetic variation within populations (Hamrick, 1992). The differentiation level within moso bamboo population was generally moderate. The Nm value of gene flow less than 1.0 is usually considered to be the threshold for significant population differentiation (Slatkin and Barton, 1989). In this study, the Nm was 3.21, indicating that there was sufficient gene flow within the population to offset the genetic differentiation caused by gene drift (Hamrick, 1992). These genetic variations contribute to the flexibility and survival of moso bamboo populations in the face of changing environmental circumstances (Olson-Manning et al., 2012). As the most important economic bamboo species, moso bamboo is likely to experience human-mediated genotype movement between distribution regions, further influencing its genetic dynamics.

### 4.3 Selection pressure on natural population of moso bamboo

Based on the analysis of selection pressure, the genetic characteristics among the subgroups of moso bamboo were identified at the molecular level. A high proportion of unique genes were identified in the three subgroups, 48.98% in α-subpopulation, 77.14% in β-subpopulation and 81.15% in γ-subpopulation. This indicates that there were different degrees of selection pressure among subgroups, which may be related to the differences in living environment, climate and human activities. Geoffrey analyzed the selection pressure of 971 sorghum plants and mapped three plant height-related genes *dw2*, *dw3* and *SbHT9.1*. These genes may represent potential variations of their main agro-climatic traits (Morris et al., 2012).

A variety of functional genes were enriched in the candidate genes screened by selection analysis, from α-subpopulation to β-subpopulation. Notably, genes related to the abscisic acid metabolism process, protein transport regulation, and supramolecular complex were enriched. Abscisic acid (ABA) is intricately involved in regulating plant dormancy, maturation and senescence, stomatal movement and various other processes (Mittler and Blumwald, 2015). Additionally, ABA plays an important role in plant responses to stressors such as drought, high salt, low temperature and pests (Hewage et al., 2020). Supramolecular complexes are essential for executing a variety of physiological functions (Croce and Amerongen, 2014). Therefore, the function of these genes may be related to the adaptability of moso bamboo, potentially influencing its response to environmental challenges and stressors.

In the process of regional differentiation from α-subpopulation to γ-subpopulation, genes related to chloroplast and chloroplast envelope were notably enriched. The area where the γ-subpopulation is located has high temperature, abundant rainfall and high humidity throughout the year. The environmental changes in the process of transmission from the central to the southern may lead to some adaptive changes in chloroplasts. Furthermore, genes associated with stimulus response and secondary cell wall were also enriched. The southern coastal areas are frequently affected by typhoons. It is speculated that these genes may regulate the growth and development of moso bamboo, enhancing the wind resistance and stress tolerance of moso bamboo.

In this study, the verification results of nonsynonymous mutation SNP loci of *PH02Gene00430* and *PH02Gene21923* related to temperature stimulus response in moso bamboo showed that HBXN and HBCY in α subgroup and JSJR in β subgroup were polymorphic. Functional analysis of these two genes revealed that their corresponding homologous genes were *XTH15* (xyloglucan endotransglucosylase/hydrolase 15) and *CRLK1* (calcium/calmodulin-regulated receptor-like kinase 1). XTHs (Xyloglucan endotransglucosylase/hydrolases) are considered chief enzymes in cell wall remodeling and play a central role in stress responses. Research shows that the content of xyloglucan components recognized by CCRC-M87/103/104/106 antibodies might be negatively related to banana chilling tolerance (Tan et al., 2024). Heat stress differentially influences the *XTH* expression profiles in the wheat seedlings (Iurlaro et al., 2016). And *CRLK1*, a calcium/calmodulin-regulated receptor-like kinase, plays an important role in regulating plant cold tolerance (Yang et al., 2010). Such as *Hosta ventricosa* perceives temperature changes via *CRLK*, and its cold tolerance is enhanced when subjected to low-temperature stress (Zhuang et al., 2021). Therefore, these two genes may be closely related to the temperature stimulus response of moso bamboo, and are potential candidate genes. The specific molecular mechanism is worthy of further study.

In the transition from the β-subpopulation to the γ-subpopulation, genes related to the defense response against bacteria were enriched in the southern group. These genes play a crucial role in enhancing plant stress resistance. The southern group, located at the southernmost end of the moso bamboo distribution, faces a growth environment characterized by high temperature, abundant rainfall, and high humidity. These conditions create a conducive breeding ground for bacteria and mildew. The genes related to defense responses against bacteria may help moso bamboo adapt to the challenging southern coastal environment.

### 4.4 Global potential distribution of moso bamboo

There are few studies on the global distribution of potential moso bamboo. SDM is widely used to statistically infer the driving factors influencing species distribution under different conservation, ecological and evolutionary scenarios, aiding in solving problems related to these processes (Zimmermann et al., 2010; Neftalí, 2011). A larger sample size and wider sampling area provide more information about the relationship between species and environment, and leading to more accurate species distribution models (Koster et al., 2014; Peterson et al., 2011; Yi et al., 2018; Yan et al., 2016).

In this study, we obtained global distribution data for moso bamboo for the first time. These data not only indicate the suitable habitats for its distribution area, but also minimize the deviation of simulation results due to the lack of sampling. By analyzing the relationship between the existence probability of moso bamboo and bioclimatic variables, we obtained their corresponding contribution rates and importance. The results showed that precipitation and temperature are the main variables affecting the distribution, which is consistent with the previous research highlighting precipitation as the key meteorological constraint factor affecting the moso bamboo distribution (Shi et al., 2020). Precipitation can impact soil moisture level, with excessively dry or wet soil conditions affecting bamboo growth and development (Koster et al., 2014). The suitable average temperature for the growth of moso bamboo is 10-20°C. When temperatures drop to a critical low temperature of -14°C, moso bamboo growth and metabolic activities will stagnate, resulting in physiological drought. With the synergistic interplay of temperature and seasonal precipitation, moso bamboo can grow vigorously.

Currently, the distribution range of moso bamboo in China is smaller than the potential range we predicted, suggesting a possible expansion in the future. Model calculations indicate that the potential distribution areas of moso bamboo globally are predominantly located in Asia, North America and Europe in the northern hemisphere. In history, moso bamboo has been introduced from China to many countries, with Japan being the only hosting contiguous moso bamboo forests. The prediction results for potential distribution area highlighted the southern region of central Japan as the core suitable area for moso bamboo (Isagi et al., 2014). These Japanese areas mainly exhibit a subtropical monsoon climate, closely resembling the climatic conditions of the southern mainland China, making them conducive the natural growth of moso bamboo. This is an important reason why it can become the main economic moso bamboo species in Japan. Consequently, this study can provide valuable insights for the international introduction of moso bamboo.

## 5 Conclusion

In this study, 193 new leaf samples were collected from 37 representative populations of moso bamboo, and the GBS sequencing was employed for the first-time genotyping of noso bamboo. The results revealed that the genetic diversity of moso bamboo population was low, and the level of heterozygosity was high. The genetic variation predominantly came from within the population. Overall, Chinese moso bamboo can be divided into three potential subgroup, central α-subpopulation, eastern β-subpopulation and southern γ-subpopulation. The α-subpopulation represents the most concentrated distribution area of modern moso bamboo in China, suggesting it may serve as the origin center of moso bamboo. Different selection pressures among the three subgroups of moso bamboo, which may be related to the adaptability of moso bamboo to water, temperature and light in the process of historical transmission, resulting in the differentiation of genetic structure among subgroups, making subgroups reflect different genetic characteristics. And the two genes (*PH02Gene00430* and *PH02Gene21923*) may be potential candidate genes related to temperature stimulus response in moso bamboo. There were no significant population expansion events in the history of Chinese moso bamboo at the level of the whole population and subgroup. However, moso bamboo experienced two bottlenecks about 8–30 ka (thousand years) and 70–120 ka years ago. In recent millennia, no substantial changes in the effective population size were detected within the three subgroups. The five dominant factors limiting the distribution of moso bamboo were the warmest season precipitation, temperature seasonality, wettest monthly precipitation, the lowest temperature of the coldest month, and annual mean temperature, with the warmest season precipitation exerting the most significant impact factor. The predictive distribution results of contemporary moso bamboo are consistent with its realistic distribution pattern. The above results provide valuable data support for the studying moso bamboo population evolution, germplasm mining, resource cultivation, and development of moso bamboo economy.

## Data Availability

The datasets presented in this study can be found in online repositories. The names of the repository/repositories and accession number(s) can be found below: https://www.ncbi.nlm.nih.gov/, PRJNA1133081.
