# Supplementary material for: Genomics reveal population structure, genetic diversity and evolutionary history of Phyllostachys edulis (moso bamboo) in global natural distribution
Source: Front Plant Sci. 2025 May 15;16:1532058. doi: 10.3389/fpls.2025.1532058 (PMC12119579; doi:10.3389/fpls.2025.1532058)
Supplement: Supplementary file 1 [file DataSheet1.docx]

**supplementary figures**

| 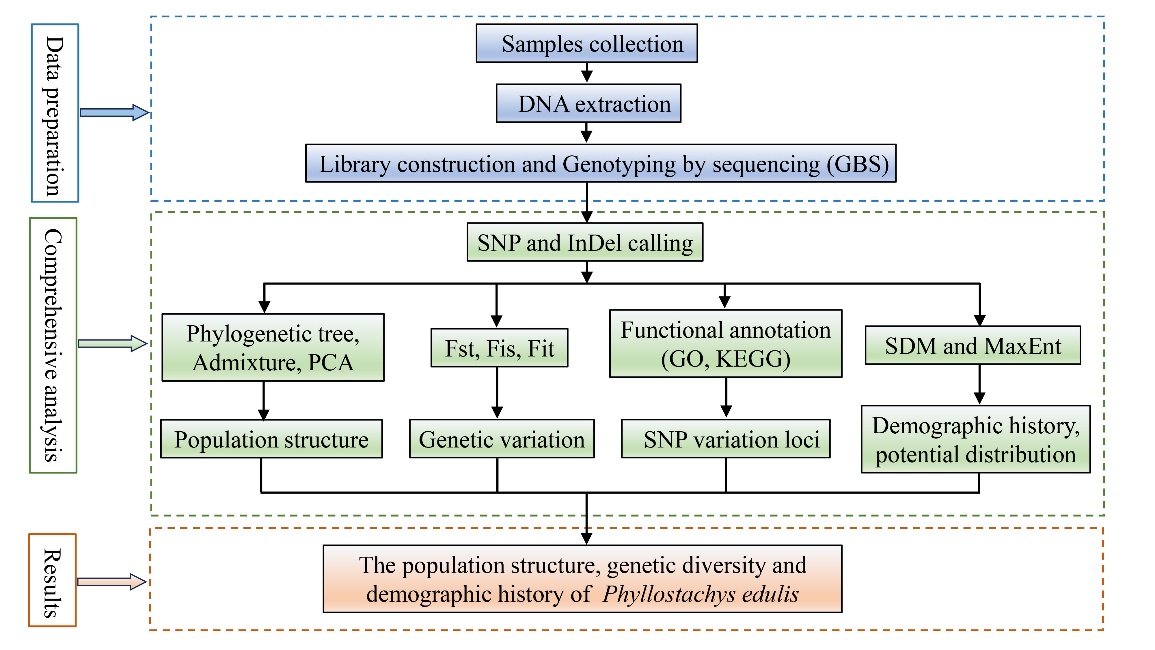 |
| --- |
| **Figure S1.** The whole study framework. |

| 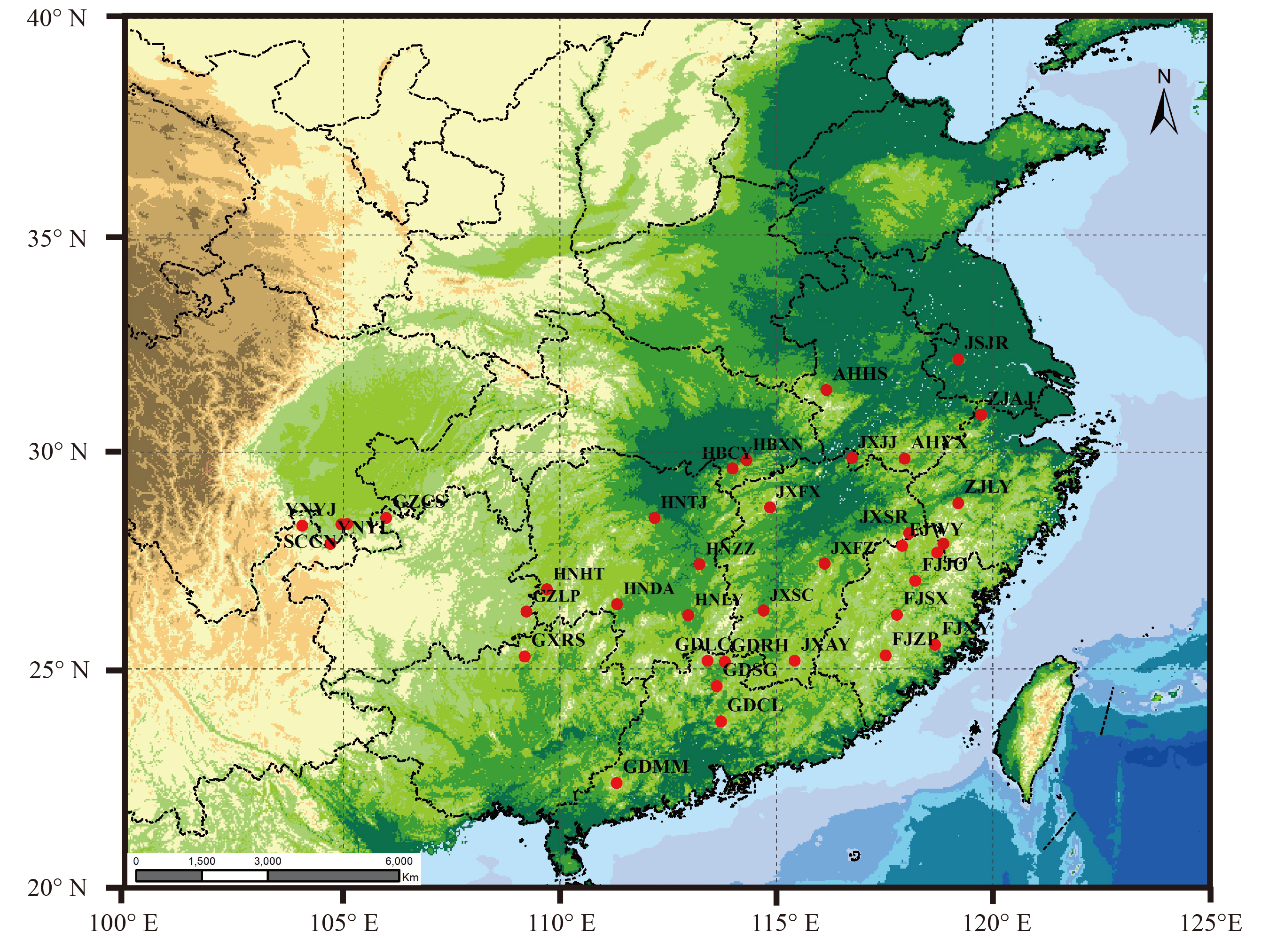 |
| --- |
| **Figure S2.** Moso bamboo sampling natural distribution. The base map quoted from ETOPO1 (<https://www.ngdc.noaa.gov/mgg/global/global.html>). |

| 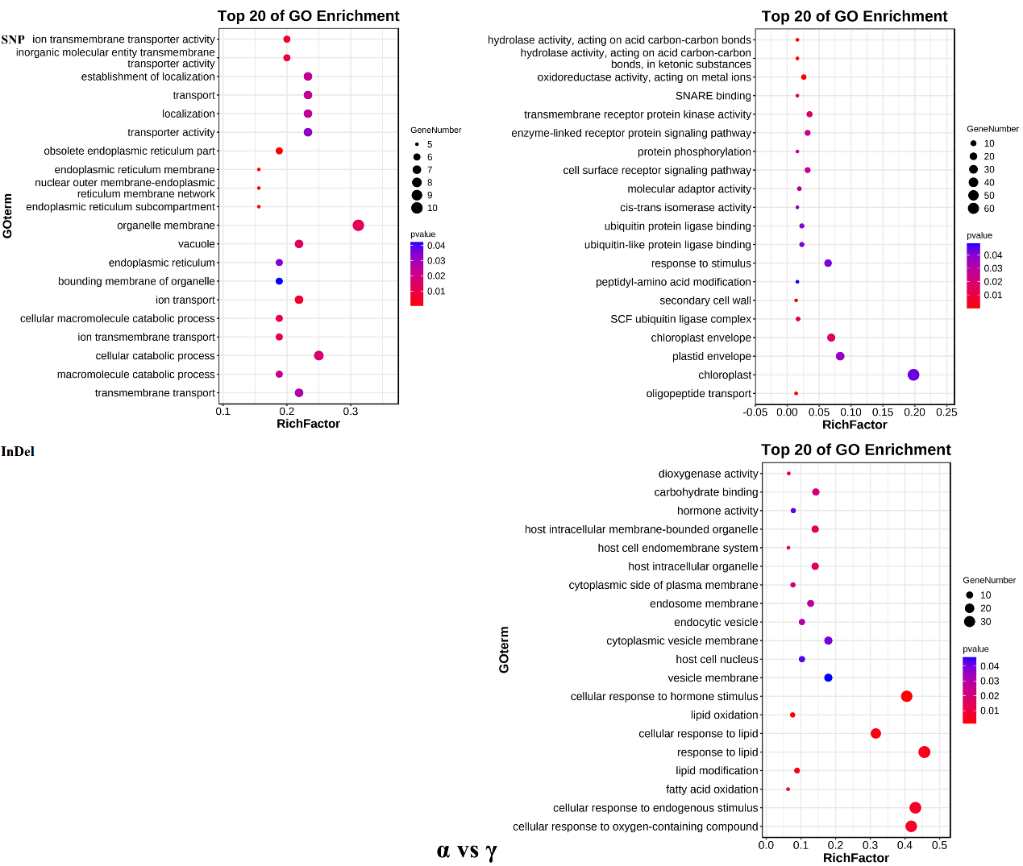 |
| --- |
| **Figure S3.** GO analysis of selected genes between α-subpopulation and γ-subpopulation. X-axis represents rich factor, y-axis represents pathway name, dots size represents gene number, and dots color represents p-value, Vacancies indicate no selection genes and no GO and KEGG analysis results. |

| 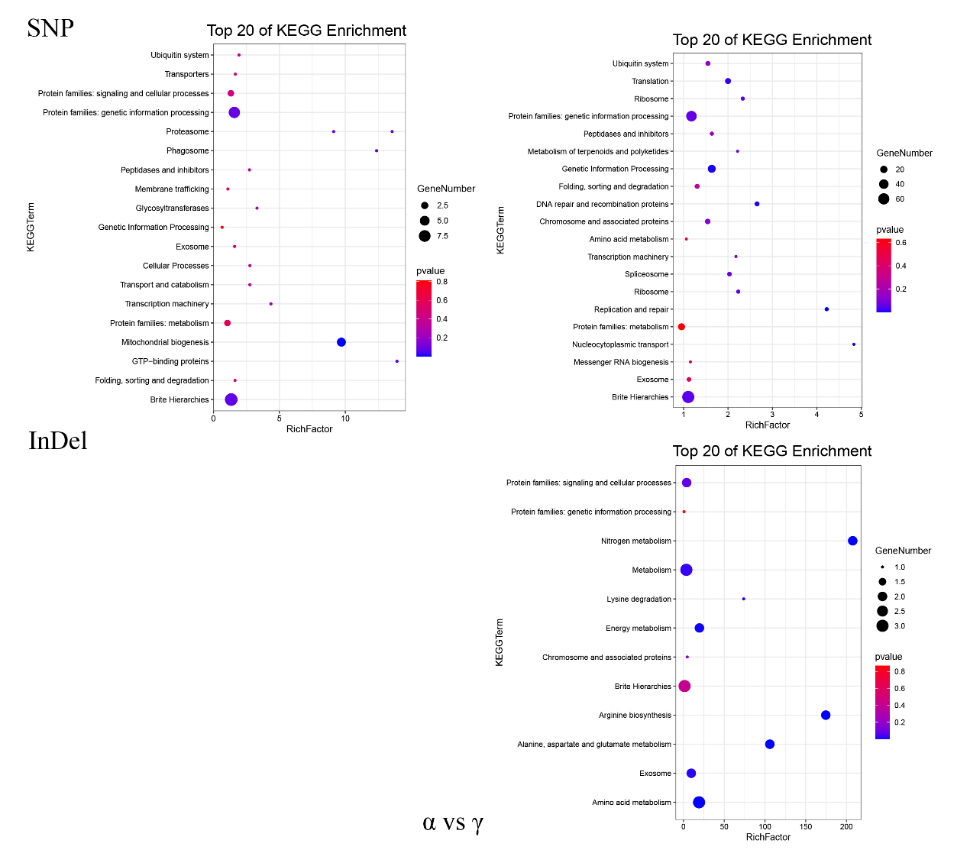 |
| --- |
| **Figure S4.** KEGG analysis of selected genes between α-subpopulation and γ-subpopulation. |

| 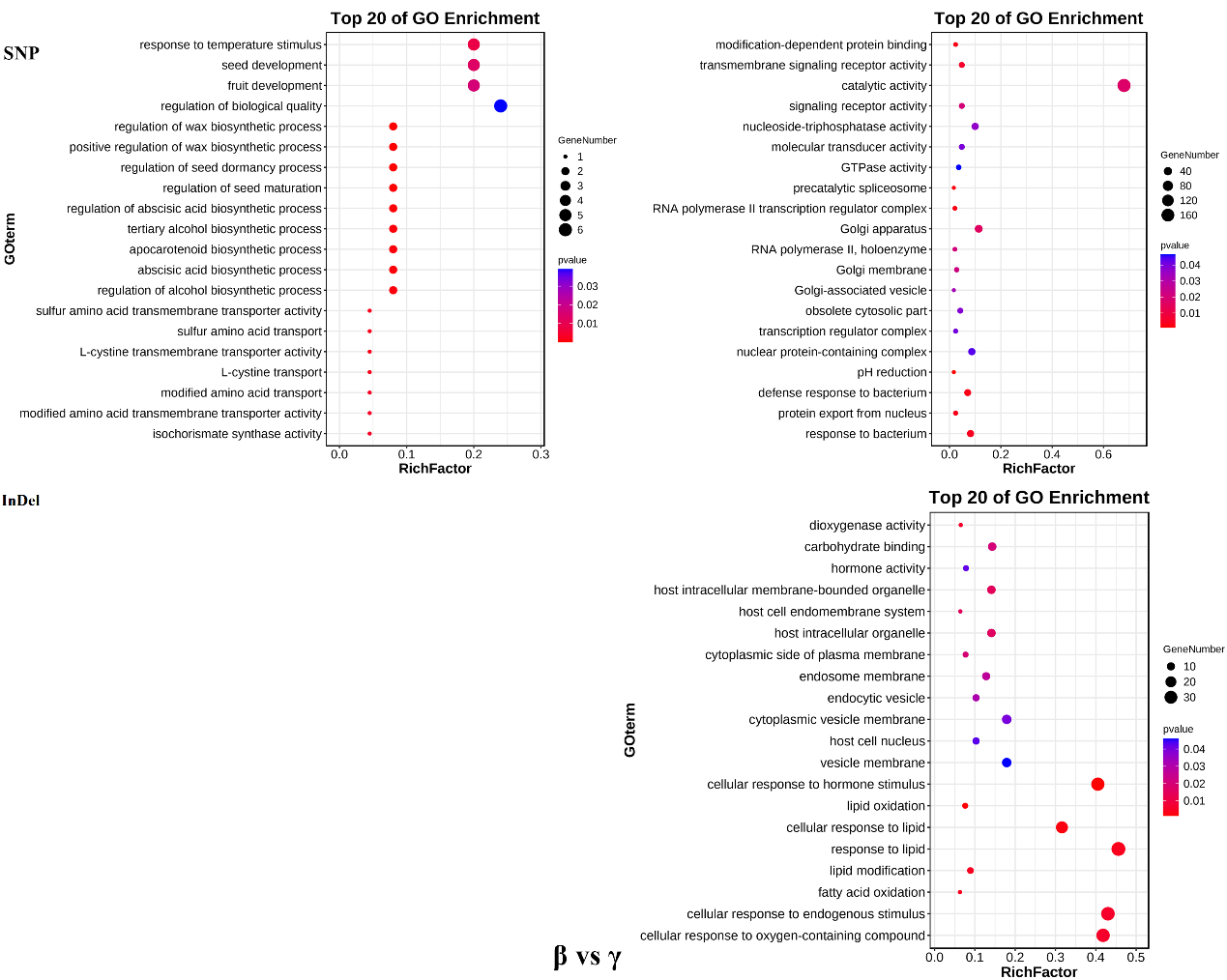 |
| --- |
| **Figure S5.** GO analysis of selected genes between β-subpopulation and γ-subpopulation. |

| 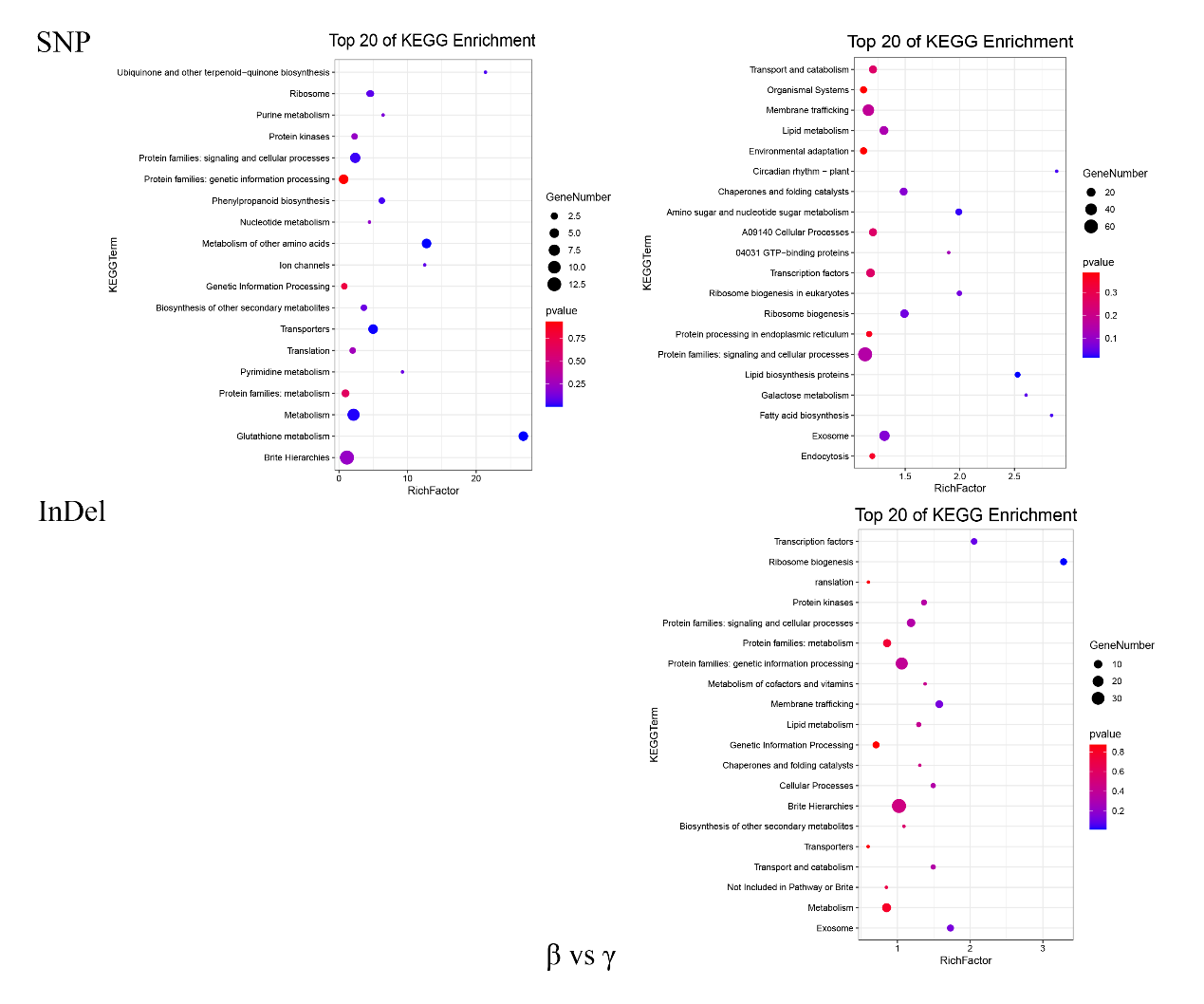 |
| --- |
| **Figure S6.** KEGG analysis of selected genes between β-subpopulation and γ-subpopulation. |

| 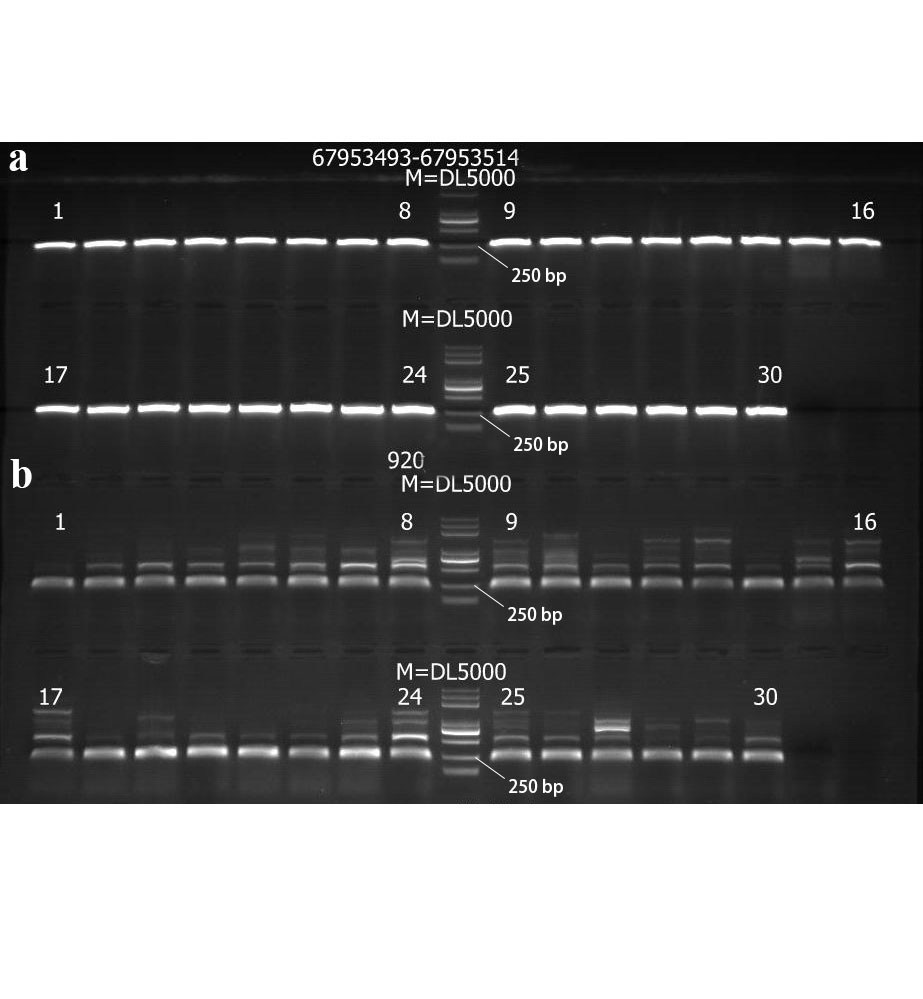 |
| --- |
| **Figure S7.** Gel electrophoresis of PCR amplification products of 30 samples. |
